# The predictive ability of the expected utility-entropy based fund rating approach: A comparison investigation with Morningstar ratings in US

**DOI:** 10.1371/journal.pone.0215320

**Published:** 2019-04-19

**Authors:** Daniel Chiew, Judy Qiu, Sirimon Treepongkaruna, Jiping Yang, Chenxiao Shi

**Affiliations:** 1 Business School, The University of Western Australia, Perth, Australia; 2 School of Economics and Management, Beihang University, Beijing, China; Institute for Economic Forecasting, Romanian Academy, ROMANIA

## Abstract

In this paper, we propose an alternative fund rating approach based on the Expected Utility-Entropy (EU-E) decision model, in which the measure of risk for a risky action was axiomatically developed by Luce et al. We examine the ability of this approach as an alternative fund rating approach for its ability to potentially mitigate the drawbacks of the risk measure used in Morningstar ratings, and investigate the ability of the EU-E model based and Morningstar ratings to predict mutual fund performance. Overall, we find that the risk measure used in both models plays a defining role in their ability to predict future fund performance, and that the EU-E model can effectively consider the behavioral decisions of an investor.

## 1 Introduction

In recent history, mutual funds have been a dominant choice among investors with the industry growing rapidly over the past 30 years and with funds managed growing from $51 billion to over $15 trillion during the period of 1976 to 2015 [1], [2]. A key driving factor behind this growth can be attributed to the vast number of U.S. investors who attempt to beat the market each year coupled with the fact that mutual funds are viewed as an economical means for investors to diversify away unsystematic risk from their portfolios [3], [4]. Due to demand and growth in the industry, simple mutual fund rating tools have been developed to assist investors in making capital allocation decisions. The most prominent agencies include Morningstar, Lippers, and Zacks, which rank funds on a scale of 1 to 5 based on a fund’s calculated risk-adjusted return. Among these fund rating approaches, Morningstar ratings play a powerful role in the mutual fund industry and are viewed as a crucial metric for investors and fund managers [5], [6]. Thus, we focus on Morningstar ratings due to its prominence in the industry and its popularity in the evaluation of fund rating systems.

Higher mutual fund ratings are an indication of better performance [7]. For fund managers, their funds’ star ratings constitute an important marketing tool used to attract investors [8]. This implication is consolidated by a recent study that substantiates the value of a fund’s star rating by providing evidence of positive abnormal flows to funds gaining stars and negative abnormal flows out of funds losing stars [9]. As investors reward funds that gain stars and penalize those that lose stars, this implies that investors believe that the rating system offers valuable information about mutual fund performance. This implication, however, has been subject to much scrutiny due to the following findings.

On one hand, Phillips et al. [10] discuss the reliance of the Morningstar rating system on holding period returns as primary performance. They investigate whether horizon effects in reported performance affect investor allocations to mutual funds. Their analysis suggests that investors fail to recognize the effect of horizons on holding period return calculations, allocating disproportionate wealth to funds. Horizon effects on performance are predictable (and thus, so are Morningstar ratings). Kaniel and Parham [11] examine the impact of media attention on capital flows. They reveal that media attention affects the consumer and mutual fund investment decisions. They show that mutual fund managers react to incentives created by the media effect in a strategic way and argue that this creates incentives to alter the risk exposure of a fund. Thus, historical risk measures are likely poor proxies for realized risk.

On the other hand, the rating system provides little predictive ability of future performance and, more notably, it is unable to differentiate between higher and median rated funds [8], [12–16]. As these ratings are used as a primary source of information in investor decision making, the inability to predict future performance by this rating system raises questions about its benefits to investors [5]. Furthermore, existing studies note that risk adjustments made in calculating fund ratings through Morningstar may not account for an appropriate proportion of risk faced by a fund [12], [17]. These findings suggest that an improvement to the Morningstar system is needed and that it would be helpful to establish an alternate funding rating approach. This inefficient adjustment for risk may be attributed in part to Morningstar’s reliance on the Expected Utility Theory (EUT), which is proven to draw outcomes that deviate from individuals’ behaviors under risk [18–20]. Additionally, risk aversion in the EUT is traditionally modeled as a concave utility function of wealth and also utilized by Morningstar ratings in calculating MRAR. Rabin [20] outlines that while theoretically sound, a concave utility function accounting for risk aversion does not account for risks faced by all prospects. This is further exemplified by findings of Niendorf and Ottaway [21] showing that market participants exhibit different risk preferences in contrasting market conditions.

Shannon [22] developed an information theory where entropy captures the uncertainty of an associated probability distribution. It has been applied to a broad body of financial literature and most prominently in studies on option pricing [23], return predictability [24], [25] and portfolio selection [26], [27]. Recent studies evaluating entropy relative to standard deviation and beta in measuring financial risk have been supportive of entropy, primarily for its distribution-free nature and ability to incorporate more information of uncertainty than the latter two measures [28–30]. This can be attributed to the fact that entropy is not bounded by assumptions of the mean-variance framework, as it is derived from the actual distribution rather than from variables and their moments [24]. Above all, it has been shown that entropy has the ability to predict market dynamics, which can be crucial in forecasting a fund’s future performance [25]. However, Shannon entropy as a measure of risk may not very adequate in capturing the overall behaviours of a risky investment given its irrelevance to the outcome of consequences of a decision-making action under risk.

Yang and Qiu [31] proposed the expected utility-entropy (EU-E) measure of risk and a decision-making model based on the expected utility and entropy of an action involving risk. The EU-E decision model brings together the notion of expected utility and entropy to create a decision model that effectively considers the decision maker’s subjective preferences and objective uncertainty at each state of nature. In addition, the EU-E model solves typical decision problems such as the Allais paradox, which is not solved by the expected utility theory. To further representations for uncertain risky actions under behavioural axioms about preference orderings among gambles and their joint receipt, Luce et al. [32] derived the numerical representations. These representations apply to uncertain alternatives and consist of a subjective utility term plus a term depending upon the events and subjective weights. For risky decision-making actions, Luce et al. [33] derived an expected utility term, plus a constant multiplies the Shannon entropy as a representation of risky choices under conditions of segregation and duplex decomposition. Their results can be taken as an axiomatic development of the EU-E model. This further demonstrates the reasonability of the EU-E decision model in Yang and Qiu [31].

In a recent study, Yang and Qiu [34] improved the EU-E model, which has certain normative properties under certain conditions. When using their model, some decision-making problems, such as the certainty effect of prospect theory, can be elucidated reasonably. Yang et al. [35] further applied the EU-E decision model to portfolios and this paper highlights the importance of using entropy when considering the uncertainty of states of nature in making decisions under risk.

In this paper, we propose an alternative rating approach tool based on the EU-E decision model [31], [34], and we evaluate it against Morningstar ratings. We contribute to the existing literature in the following way.

We propose a fund rating approach based on the EU-E model to potentially mitigate the drawbacks of the risk measure used in Morningstar ratings, stemming from its inadequacy in capturing risk. To date, the work by Bechmann and Rangvid [36] on a cost-based rating system, the atpRating, is the only study examining the performance of Morningstar ratings relative to an alternative model. The atpRatings are limited to Danish funds and thus provides a limited scope considering the relative size of the Danish mutual fund industry. Our study investigate the ability to predict mutual fund performance of Morningstar ratings and the EU-E model on the U.S. market, globally the largest mutual fund industry.

## 2 Methodology

### 2.1 Fund ratings approach based on the EU-E decision model

We propose the fund rating approach based on the EU-E decision model [31], [34]. Firstly, the probability distribution of monthly returns of each fund is constructed. Next, we calculate entropy, expected utility, and risk by using the probability distribution at the end of each month. The net expected utility yielded by each fund is then calculated by subtracting a fund’s the EU-E measure of risk from its expected utility. For each month, the funds are ranked by their net expected utility, where a higher value is better. Finally, the funds are sorted into categories of 1 to 5 stars, from which we can calculate the funds’ overall ratings using the same approach as Morningstar. This process can be demonstrated more formally as follows.

We consider a sample of *m* funds and denote the action of selecting fund $S_{i}$ as $a_{i}$ (*i* = 1, 2,…, *m*). Firstly, for each fund, we collect the return of *l* previous months and denote the return series of fund Si's returns as $r_{i1},r_{i2},\cdots ,r_{il}$ and $a=\underset{1\leq i\leq m}{\min}\{r_{i1},r_{i2},\cdots ,r_{il}\}$, $b=\underset{1\leq i\leq m}{\max}\{r_{i1},r_{i2},\cdots ,r_{il}\}$, to form an interval [*a*, *b*]. Next, we create the distribution of monthly returns by dividing interval [*a*, *b*] into *n* equal sub-intervals $\left[r_{0},r_{1}\right)$, $\left[r_{1},r_{2}\right)$,…, $\left[r_{n-1},r_{n}\right]$, where $a=r_{0}<r_{1}<\ldots <r_{n}=b$, denoting these intervals as $\theta_{j}$ (*j* = 1, 2,…, *n*), respectively. In this study, we divide the monthly return distribution into 11 equal sub-intervals similar to Yang et al [35]. We then calculate the frequency of fund Si's return which falls within interval $\theta_{j}$, denoted by $\rho_{ij}$, and let the expected return in interval $\theta_{j}$ of $S_{i}$ be ${\bar{x}}_{ij}$. According to the law of large numbers by Bernoulli, $\rho_{ij}$ approaches the probability distribution of the sample, $p_{ij}$, as *l* increases. Therefore, if *l* is large enough, $\rho_{ij}$ can be regarded as an approximation of $p_{ij}$. Thus, this forms the probability distribution for each fund at each particular month. Consequently, we can assume that in the future, fund Si's return takes an expected value of ${\bar{x}}_{ij}$ within interval $\theta_{j}$ drawn from the probability distribution, $p_{ij}$.

From the probability distribution, we can then calculate entropy of the distribution corresponding to action $a_{i}$ as:

$$H_{a_{i}}(\theta )=-\sum_{i=1}^{n}\rho_{ij}\text{ln}\rho_{ij}$$ (1)

Additionally, the normalized expected utility of risky action $a_{i}$ is:

$$E\left[u(a_{i})\right]=\frac{\sum_{j=1}^{n}\left[u({\bar{x}}_{ij})\rho_{ij}\right]}{\underset{1\leq i\leq m}{\max}\sum_{j=1}^{n}\left[u({\bar{x}}_{ij})\rho_{ij}\right]}$$ (2)

Combining the normalized measures of Eqs (1) and (2) weighted by the investor’s respective risk preference (denoted by the trade-off coefficient, λ), then the EU-E measure of risk is defined as:

$$R(a_{i})=-\lambda \sum_{j=1}^{n}\rho_{ij}\ln\rho_{ij}/\ln(n)-(1-\lambda )\sum_{j=1}^{n}\left[u({\bar{x}}_{ij}\right)\rho_{ij}]/\underset{1\leq i\leq m}{\max}\{⥄|\sum_{j=1}^{n}u({\bar{x}}_{ij})\rho_{ij}|\}$$ (3)

where $R(a_{i})$ is the EU-E risk measure of investing fund Si(i=1,2,…,m).

Firstly, we calculate the EU-E risk measure of investing a fund during a specified period using Eq (3).

Next, we define the net normalized expected utility yielded by each fund which is calculated by subtracting a fund’s risk from its normalized expected utility. That is, using Eqs (2) and (3), we can calculate each fund’s net normalized expected utility for each month by taking a fund’s normalized expected utility from its risk shown in Eq (4). We can then rank all the funds in each month by their normalized net expected utility, where a higher value is better.

$$NetE[u(a_{i})]=\sum_{j=1}^{n}\left[u({\bar{x}}_{ij})p_{ij}\right]/\underset{1\leq i\leq m}{\max}\left\{\left|\sum_{j=1}^{n}\left[u({\bar{x}}_{ij})p_{ij}\right]\right|\right\}-R(a_{i})$$ (4)

Finally, we apply the above procedure to the funds’ past 3-, 5- and 10-year monthly returns to obtain their respective 3-, 5- and 10-year ratings throughout the sample. And then, we obtain each fund’s overall rating using Morningstar’s “50:30:20” approach at each month, resulting in our monthly fund rankings based on the EU-E model [37]. Then, the funds are categorized into ratings of 1 to 5 stars, that is, the top 10% of funds receive a 5-star rating, the next 22.5% receive a 4-star rating, the next 35% attain a 3-star rating, the following 22.5% receive a 2-star rating and the remaining 10% are given a 1-star rating [37].

### 2.2 Out-of-sample performance measures

To test the predictive power of the fund ratings based on Morningstar and the EU-E model, we perform a panel regression analysis where the dependent variable is the out-of-sample performance measure. We adopt four out-of-sample performance measures: the Sharpe ratio, Jensen’s alpha, and the alpha estimated from the Fama-French 3-factor and Carhart 4-factor models. It follows that the Sharpe ratio measures the reward per unit of total risk, however, the alpha estimated from the single-, three- and four-factor models measures fund performance by adjusting for risk. In calculating each measure, the returns of a fund measured three years prior to the observation date are used [14], [16]. These four out-of-sample performance measures are defined in the following.

The Sharpe [38] ratio is defined as follows:

$$Sharpe_{i,t}=\frac{\bar{R_{i}-R_{F}}}{\sigma_{i}}$$ (5)

where $\bar{R_{i}-R_{F}}$ is the excess return of fund *i* and $\sigma_{i}$ is standard deviation of monthly returns of fund *i* over the estimation period of three years prior to time *t*.

Jensen’s alpha is estimated from the Capital Asset Pricing Model (CAPM):

$$R_{it}-R_{ft}=\alpha_{i}+\beta_{i}(R_{mt}-R_{ft})+\varepsilon_{it}$$ (6)

where $R_{it}$ is the return of fund *i* in month *t*, $R_{mt}$ refers to the return of the S&P 500 in month *t*, $\alpha_{i}$ is the alpha for fund *i*, $\beta_{i}$ is the sensitivity of fund *i*’s excess return to the S&P 500 index, and $\varepsilon_{it}$ is the error term for fund *i* at time *t*.

The alpha from the Fama-French [39] 3-factor model is estimated as follows:

$$R_{it}-R_{ft}=\alpha_{i}+\beta_{i}(R_{mt}-R_{ft})+s_{i}SMB+h_{i}HML+\varepsilon_{it}$$ (7)

where *SMB* (small minus big) refers to the size factor and *HML* (high minus low) refers to the value factor.

The Carhart [40] 4-factor model introduces momentum as an additional factor into the original Fama-French 3-factor model. The alpha from the 4-factor model is estimated using the following model:

$$R_{it}-R_{ft}=\alpha_{i}+\beta_{i}(R_{mt}-R_{ft})+s_{i}SMB+h_{i}HML+m_{i}UMD+\varepsilon_{it}$$ (8)

where *UMD* (up minus down) refers to the momentum factor.

### 2.3 Panel regression model

Prior studies evaluating the predictive ability of Morningstar ratings adopt cross-sectional dummy variable regressions in estimating the model [8, 14, 16, 41]. However, cross-sectional data only considers variables at one particular point in time whereas panel data combines cross sectional regressions with time series together. Thus, we use panel regressions to investigate the performance of the models over the sample periods. To test the predictive ability of ratings based on Morningstar and the EU-E model, we adopt and estimate the following panel regression model:

$$S_{i,t+1}=\delta_{5}+\delta_{4}D4_{it}\text{+}\delta_{3}D3_{it}+\delta_{2}D2_{it}+\delta_{1}D1_{it}+\varepsilon_{it}$$ (9)

where $S_{i,t+1}$ is the out-of-sample performance measure for fund *i. $D1_{it}$, $D2_{it}$*, $D3_{it}$ and $D4_{it}$ are dummy variables set at 1 when fund *i* receives a 1-star, 2-star, 3-star or 4-star overall rating and set at 0 otherwise, respectively. The coefficient $\delta_{5}$ corresponds to the performance of a fund with a 5-star overall rating. As such, the coefficients estimated for the remaining dummy variables indicate the performance of the particular star category relative to the 5-star category. Thus, when the performance measure is accurate in its predictive ability, better performing funds are rated higher and we should observe an increasingly negative relationship among the coefficients from $\delta_{4}$ to $\delta_{1}$ such that $0>\delta_{4}>\delta_{3}>\delta_{2}>\delta_{1}$.

This paper estimates panel data models through a rolling window analysis in each of the examined sub-samples. For panel data models, it is important to adopt an appropriate form. There are three main static panel data models: the pooled regression, fixed effect and random effect models. We use *F* and Hausman tests to determine the form of the regression model.

An *F*-test is used to determine whether the model is a pooled regression model or a fixed-effect model. The null hypothesis states that regression coefficients estimated using the pooled regression model are more efficient than the fixed effect model. The *F* statistic is

$$F=\frac{\left(R_{ur}^{2}-R_{r}^{2}\right)/\left(N-1\right)}{R_{r}^{2}/\left(n-N-k\right)}$$ (10)

where $R_{ur}^{2}$ and $R_{r}^{2}$ are the residual sum of squares of the pooled regression model and of the fixed effect model, respectively, *n* is the number of observations, *N* is the number of individuals in the section, and *k* is the number of parameters to be estimated. When the *F* statistic is significant, the fixed effect model should be used.

A Hausman test is conducted to determine whether the model is a random effect model or a fixed effect model. The null hypothesis states that both the Least Square Dummy Variable (LSDV) estimator ${\hat{\beta }}_{w}$ and the generalized least-squares estimator ${\hat{\beta }}_{GLS}$ are consistent and that the LSDV estimator is not valid. Therefore, under the null hypothesis, the difference between two estimators should not be large and should narrow as the sample increases and gradually approach zero. The Hausman statistic is

$$W=({\hat{\beta }}_{w}-{\hat{\beta }}_{GLS}{)}^{\prime }{\text{(}Var{\hat{\beta }}_{w}-Var{\hat{\beta }}_{GLS})}^{-1}({\hat{\beta }}_{w}-{\hat{\beta }}_{GLS})$$ (11)

where $Var{\hat{\beta }}_{w}$ and $Var{\hat{\beta }}_{GLS}$ are the covariance matrixes of the two estimators.

Under the null hypothesis, this statistic is asymptotically distributed as a central chi-square with *K* degrees of freedom. When the null hypothesis is true, a random effect model is used.

## 3 Data and correlation of fund performance measures

### 3.1 Data and descriptive statistics

As U.S. mutual funds compose the largest share of the global industry, amassing net assets in excess of $15.7 trillion, U.S. mutual funds are chosen in this study [2].

We retrieve monthly return and overall rating data for all U.S. mutual funds over the period of August 1992 to July 2015 from the Morningstar Direct database. Funds not assigned an overall rating or with missing data points over the 23-year period are excluded to ensure that ratings based on the EU-E model are comparable to those of the Morningstar database. There is an asymmetry in the ratings of seasoned funds that translates into a bias in the overall ratings of these funds that is absent in young funds [42]. The age bias present is attributed to the weighting system used, the climate of the evaluation period and the fund sizes. This age bias is also observed from the new Morningstar ratings methodology introduced in 2002 [6]. To eliminate the age bias shown by Morey [42], we exclude funds without 10 years of data prior to August 2002. This results in a final sample of 2,159 U.S. mutual funds. All returns are also winsorized at the 1% level.

Furthermore, to calculate out-of-sample performance measures, we obtain monthly data for 90-day U.S. T-Bill rates drawn from the FRED, market risk premiums, SMB, HML, and MOM from Kenneth French’s website over the period August 1992 to July 2015. All data obtained from Morningstar and Kenneth French’s website comply with the terms of service of the respective sources.

As previously mentioned, our overall out-of-sample period spans from August 2002 to July 2015. We partition the overall sample period into one-, three-, and five-year sub-samples such that each evaluation period comprises the ratings recorded at each month over the sample period to assess their near-, medium-, and long-term predictive abilities, respectively.

To examine the effect of excessive volatility, we further partition our sample into crisis vs non-crisis periods. Our financial crisis periods include the two most prominent market shocks within our sample period: The Global Financial Crisis (GFC) which spans the period August 2007 to February 2011 and the European Debt Crisis (EDC) comprising the period March 2010 to September 2011. Furthermore, to investigate the impact of economic recessions on our analysis, we further partition our sample into recession vs. non-recession periods. The recession period is defined as the period from December 2007 to June 2009. Table 1 reports all sample periods used in our analysis.

**Table 1:** Summary of sub-sample periods.

	Out-of-Sample Period	*N*	*M*	Total Obs.
**Panel A:** 1-Year Sub-Samples				
1	08/2002–07/2003	2159	12	25,908
2	08/2004–07/2005	2159	12	25,908
3	08/2006–07/2007	2159	12	25,908
4	08/2012–07/2013	2159	12	25,908
5	08/2014–07/2015	2159	12	25,908
**Panel B:** 3-Year Sub-Samples				
1	08/2002–07/2005	2159	36	77,724
2	08/2004–07/2007	2159	36	77,724
3	08/2006–07/2009	2159	36	77,724
4	08/2010–07/2013	2159	36	77,724
5	08/2012–07/2015	2159	36	77,724
**Panel C:** 5-Year Sub-Samples				
1	08/2002–07/2007	2159	60	129,540
2	08/2004–07/2009	2159	60	129,540
3	08/2006–07/2011	2159	60	129,540
4	08/2008–07/2013	2159	60	129,540
5	08/2010–07/2015	2159	60	129,540
**Panel D:** Crisis Sub-Samples				
GFC	08/2007–02/2009	2159	19	41,021
EDC	03/2010–09/2011	2159	19	41,021
**Panel E:** Recession Sub-Sample				
Recession	12/2007–06/2009	2159	19	41,021

*Note*: *N* refers to the number of funds considered at each month in the sample period, *M* represents the number of months considered in the sample period, Total Obs. refers to the total number of observations in the sample period (i.e. *N* x *M*).

To decipher the characteristics of our sample, Table 2 reports summary statistics for the overall sample period, the GFC, EDC, and economic recession periods. As shown in Table 2, the mean return of the funds hits its lowest level of -1.31%, while the standard deviation of fund returns records its highest value of 1.31% during the Global Financial Crisis (GFC) period. This is followed by the U.S. recession and the European Debt Crisis (EDC) period. We find a greater impact of the U.S. recession than the EDC for several reasons. Firstly, the U.S. recession is an internal shock to the U.S. market, while the EDC is an external shock with an indirect impact on the U.S. economy. A few specific events such as the announcement of the Greek bailout and speculations of defaults by vulnerable European nations had some effects on the U.S. market. Secondly, the recession coincided with the GFC, explaining the relatively similar characteristics of the two sample periods. Next, we find that monthly returns during the overall sample period yield skewness of 0.29 and kurtosis of -0.87, deviating from the assumptions of a normal distribution for fund returns. This is confirmed by the highly significant Jacque-Bera test statistic of 1378.10. The non-normal distribution is consistent across the GFC, EDC, and recession samples.

**Table 2:** Descriptive statistics of monthly returns and Sharpe ratios for US mutual funds.

	Overall Sample	GFC	EDC	Recession
*N*	336,804	41,021	41,021	41,029
Mean	0.58	-1.31	0.38	-0.7
S.D.	0.25	1.31	0.3	0.85
Skewness	0.29	-0.09	0.69	-0.19
Kurtosis	-0.87	-1.44	5.72	-1.4
Min.	0.05	-5.46	-1.39	-3.7
Med.	0.58	-1.25	0.4	-0.61
Max.	1.46	1.07	2	0.82
Jacque-Bera	1378.10*	1773.32*	820.12*	1750.27*

*Note*: The total number of observations *N* is defined as the number of funds at each period in the sample period multiplied by the number of months included in the sample period. * indicates significance at the 1% level.

### 3.2 Correlation of fund performance measures

Eling and Schuhmacher [43] find that all performance measures for evaluating investment funds, including the Sharpe’s, Jensen’s measures, as well as the excess return on value at risk, the conditional Sharpe ratio etc., display a very high rank correlation with respect to the Sharpe ratio as well as in relation to each other. Similarly to Eling and Schuhmacher [43], we analyse the Pearson correlations of the out-of-sample performance measures used in this study shown in Table 3. Panel A of Table 3 presents the correlations of the four measures for the overall sample period. We find positive correlations among the performance measures. In particular, strong positive correlations are detected among Jensen’s alpha, the alpha from the Fama-French 3-factor and Carhart 4-factor models. For example, the correlation between Jensen’s alpha and the alpha from the Fama-French 3-factor model is 0.872, and that between alphas from both the Fama-French 3-factor and Carhart 4-factor models is almost perfect at 0.971. However, the Sharpe ratio weakly correlates to the alpha from the factor models.

**Table 3:** Correlations of out-of-sample performance measures.

Panel A: Correlations during Overall Sample			
	Sharpe Ratio	Jensen’s Alpha	Fama-French 3-Factor
Jensen’s Alpha	0.379*		
Fama-French 3-Factor	0.394*	0.872*	
Carhart 4-Factor	0.403*	0.851*	0.971*
Panel B: Correlations during GFC			
	Sharpe Ratio	Jensen’s Alpha	Fama-French 3-Factor
Jensen’s Alpha	0.574*		
Fama-French 3-Factor	0.569*	0.970*	
Carhart 4-Factor	0.515*	0.886*	0.901*
Panel C: Correlations during EDC			
	Sharpe Ratio	Jensen’s Alpha	Fama-French 3-Factor
Jensen’s Alpha	0.524*		
Fama-French 3-Factor	0.514*	0.882*	
Carhart 4-Factor	0.441*	0.832*	0.958*
Panel D: Correlations during Recession			
	Sharpe Ratio	Jensen’s Alpha	Fama-French 3-Factor
Jensen’s Alpha	0.598*		
Fama-French 3-Factor	0.593*	0.982*	
Carhart 4-Factor	0.517*	0.887*	0.893*

*Note*: * indicates statistical significance at the 1% level.

The relationships observed in the overall sample period are relatively unchanged when evaluating the correlations during the GFC, EDC, and recession periods. In each period, the correlations between Jensen’s alpha and the alpha from the three- and four-factor models remains strong and positive. Interestingly, a stronger correlation between the Sharpe ratio and the alphas from the single-, three- and four-factor models are observed during the crisis and recession periods. Overall, these relationships suggest that the information provided by each of the performance measures is relatively similar, particularly from Jensen’s alpha and the alpha from the three- and four-factor models. So the regression results that the Sharpe ratio is listed as explanatory variables are representative.

## 4 Predictive ability of the fund ratings approaches

### 4.1 Predictive ability of fund ratings in the overall sample period

#### 4.1.1 Predictive ability of Morningstar ratings

In order to test the predictive ability of the fund rating approach based on the EU-E decision model, we first examine the predictive ability of Mornings ratings, and then compare the predictive ability of both rating methods.

We present panel regression results derived from Morningstar ratings where the out-of-sample performance measure is the Sharpe ratio. The Sharpe ratio is calculated one month in advance of the published ratings using the returns for each fund measured 3 years prior to the month evaluated. Each sub-sample period begins in August and ends in July of the denoted year. Clustered standard-errors are reported in parentheses. Year fixed-effects are included. Panels A, B, and C illustrate the one-, three-, and five-year sub-samples, respectively.

Table 4 reports the results of the panel regressions for Morningstar ratings over each sub-sample period while using the Sharpe ratio as the dependent variable. The coefficient estimate, $\delta_{5}$, can be thought of as the average reward per unit of total risk earned by 5-star funds for each sub-sample period. This coefficient is generally positive and significant, indicating that Morningstar’s 5-star rated funds produce a positive risk-adjusted return on average. The coefficient of the 5-star category is negative and insignificant on one occasion: for the 2008–2013 five-year sub-sample. However, we do not observe a negative and insignificant coefficient on $\delta_{5}$ for the 2008–2013 sub-sample for the other remaining three out-of-sample measures.

**Table 4:** Panel regression results derived from Morningstar ratings where the Sharpe ratio measures out-of-sample Performance.

	$\delta_{5}$	$\delta_{4}$	$\delta_{3}$	$\delta_{2}$	$\delta_{1}$	$\mathrm{Adj}.R^{2}$	Obs.
**Panel A:** One-Year Sub-Samples							
2002–2003	0.132***	-0.109***	-0.113***	-0.119***	-0.166***	0.04	25,908
	(0.015)	(0.017)	(0.017)	(0.019)	(0.023)		
2004–2005	0.267***	-0.048***	-0.071***	-0.109***	-0.171***	0.12	25,908
	(0.007)	(0.008)	(0.008)	(0.009)	(0.020)		
2006–2007	0.336***	-0.058***	-0.108***	-0.175***	-0.257***	0.12	25,908
	(0.012)	(0.013)	(0.013)	(0.014)	(0.024)		
2012–2013	0.419***	-0.067***	-0.094***	-0.119***	-0.159***	0.06	25,908
	(0.013)	(0.014)	(0.013)	(0.015)	(0.017)		
2014–2015	0.440***	-0.030**	-0.065***	-0.097***	-0.298***	0.06	25,908
	(0.011)	(0.012)	(0.012)	(0.013)	(0.093)		
**Panel B:** Three-Year Sub-Samples							
2002–2005	0.113***	-0.077***	-0.089***	-0.111***	-0.164***	0.15	77,724
	(0.010)	(0.010)	(0.010)	(0.011)	(0.016)		
2004–2007	0.275***	-0.040***	-0.080***	-0.135***	-0.206***	0.15	77,724
	(0.009)	(0.009)	(0.009)	(0.010)	(0.019)		
2006–2009	0.326***	-0.053***	-0.102***	-0.155***	-0.223***	0.43	77,724
	(0.008)	(0.008)	(0.008)	(0.009)	(0.011)		
2010–2013	0.179***	-0.056***	-0.082***	-0.100***	-0.140***	0.29	77,724
	(0.011)	(0.012)	(0.012)	(0.013)	(0.015)		
2012–2015	0.410***	-0.048***	-0.080***	-0.113***	-0.207***	0.07	77,724
	(0.010)	(0.008)	(0.008)	(0.010)	(0.034)		
**Panel C:** Five-Year Sub-Samples							
2002–2007	0.114***	-0.062***	-0.087***	-0.127***	-0.184***	0.19	129,540
	(0.008)	(0.008)	(0.008)	(0.008)	(0.013)		
2004–2009	0.280***	-0.044***	-0.087***	-0.138***	-0.206***	0.47	129,540
	(0.007)	(0.007)	(0.008)	(0.008)	(0.011)		
2006–2011	0.319***	-0.057***	-0.097***	-0.136***	-0.195***	0.37	129,540
	(0.008)	(0.008)	(0.008)	(0.008)	(0.009)		
2008–2013	-0.009	-0.061***	-0.088***	-0.110***	-0.155***	0.57	129,540
	(0.009)	(0.010)	(0.010)	(0.011)	(0.012)		
2010–2015	0.179***	-0.049***	-0.078***	-0.103***	-0.176***	0.19	129,540
	(0.009)	(0.008)	(0.008)	(0.009)	(0.024)		

*N*ote: Clustered standard-errors are reported in parentheses. Year fixed-effects are included. ** and *** indicate statistical significance at the 5% and 1% levels, respectively.

A negative coefficient on each dummy variable represents an underperformance of each star category relative to the 5-star category. For example, the coefficient on $\delta_{4}$ of -0.109 in the 2002–2003 sub-sample indicates that on average 4-star funds earned a risk-adjusted return which is 10.9% less than that of 5-star funds during the 2002–2003 sub-sample. Furthermore, coefficients $\delta_{\text{3}}$ of -0.113, $\delta_{\text{2}}$ of -0.119, and $\delta_{\text{1}}$ of -0.166 exhibit an increasingly negative relationship such that on average the 4-star funds outperform the 3-star funds, which also outperform the 2-star funds and so on. This indicates that Morningstar ratings offer superior predictive ability. This increasingly negative relationship from $\delta_{4}$ to $\delta_{\text{1}}$ is consistent across the one-, three-, and five-year sub-samples and across all out-of-sample performance measures. Hence, the predictive ability of Morningstar ratings extends from the near- to long-term regardless of the out-of-sample performance measure applied.

Our findings contrast with those of most existing studies on the predictive ability of Morningstar ratings [8] [13–16]. Of existing studies, Blake and Morey [8] examine the predictive ability of Morningstar’s old rating methodology for the U.S. as opposed to Morningstar’s new methodology, which is examined in our study (Morningstar’s new approach applies to fund ratings published from July 2002 onwards, while its old methodology applies to fund ratings published prior to July 2002). Although Füss et al. [14], Sah et al. [15], and Watson et al. [16] examine the new methodology, they conduct their studies on regions outside of the U.S. and on specific sectors. Further, our finding that Morningstar ratings offer superior predictive ability among star rating groups is consistent with Morey and Gottesman [41], who adopt Morningstar’s new method and find that Morningstar ratings can predict future fund performance using a sample of U.S. mutual funds in a 3-year sample period from July 2002 to June 2005.

#### 4.1.2 Predictive ability of ratings based on EU-E (λ = 0.25)

Before applying the fund ratings based on EU-E model to rank all 2159 funds, we need to determine investors’ utility function *u*(*x*). For the sake of simplicity, we use a linear function of utility in the paper. To test predictive ability of the fund rating approach based on EU-E decision model, firstly, we perform the fund ratings based on EU-E model when *λ* = 0.25 on the all 2159 funds at each month during different sample periods. The reason to chose *λ* = 0.25 is that it is the first quartile of the range of *λ* which is the appropriate trade-off between the expected utility and entropy indicating a lower weight in the uncertainty of the return of the fund, and vice versa.

We present the results of the panel regressions for ratings based on EU-E (*λ* = 0.25) where the Sharpe ratio proxies for the out-of-sample performance are measured. The Sharpe ratio is calculated one month in advance of the published ratings using the returns for each fund derived 3 years prior to the month evaluated. Each sub-sample period begins in August and ends in July of the denoted year. Clustered standard-errors are reported in parentheses. Year fixed-effects are included. Panels A, B, and C illustrate the one-, three-, and five-year sub-samples, respectively.

Overall, our results support the EU-E model as a predictor of future fund performance. Firstly, the coefficient, $\delta_{5}$, is positive and significant across all sub-sampled periods. This suggests that on average the 5-star group of ratings based on EU-E (*λ* = 0.25) earns positive risk-adjusted returns across all sub-sample periods. Furthermore, the regressions consistently present a significant and increasingly negative relationship from $\delta_{4}$ to $\delta_{\text{1}}$, which implies that ratings based on the EU-E model where *λ* is 0.25 offer significant predictive ability of future fund performance. It is also interesting to note that the magnitudes of $\delta_{5}$ reported in Table 5 are higher than those reported in Table 4. This implies that the risk-adjusted return earned by the EU-E model outperforms Morningstar’s 5-star funds.

**Table 5:** Panel regression results derived from ratings based on EU-E (*λ* = 0.25) where the Sharpe ratio measures out-of-sample performance.

	$\delta_{5}$	$\delta_{4}$	$\delta_{3}$	$\delta_{2}$	$\delta_{1}$	$\mathrm{Adj}.R^{2}$	Obs.
**Panel A:** One-Year Sub-Samples							
2002–2003	0.261***	-0.087***	-0.205**	-0.377***	-0.508***	0.38	25,908
	(0.010)	(0.011)	(0.012)	(0.013)	(0.013)		
2004–2005	0.274***	-0.034***	-0.067***	-0.111***	-0.176***	0.19	25,908
	(0.006)	(0.007)	(0.008)	(0.007)	(0.010)		
2006–2007	0.465***	-0.155***	-0.258***	-0.293***	-0.449***	0.35	25,908
	(0.009)	(0.010)	(0.010)	(0.011)	(0.015)		
2012–2013	0.415***	-0.065***	-0.101***	-0.123***	-0.100***	0.06	25,908
	(0.007)	(0.007)	(0.008)	(0.009)	(0.020)		
2014–2015	0.521***	-0.040***	-0.137***	-0.245***	-0.339***	0.15	25,908
	(0.006)	(0.006)	(0.007)	(0.007)	(0.041)		
**Panel B:** Three-Year Sub-Samples							
2002–2005	0.167***	-0.056***	-0.124***	-0.215***	-0.316***	0.33	77,724
	(0.007)	(0.007)	(0.008)	(0.009)	(0.010)		
2004–2007	0.376***	-0.126***	-0.189***	-0.215***	-0.317***	0.27	77,724
	(0.007)	(0.007)	(0.008)	(0.008)	(0.011)		
2006–2009	0.420***	-0.130***	-0.215***	-0.244***	-0.331***	0.53	77,724
	(0.006)	(0.006)	(0.006)	(0.007)	(0.009)		
2010–2013	0.218***	-0.069***	-0.127***	-0.172***	-0.152***	0.32	77,724
	(0.005)	(0.004)	(0.006)	(0.006)	(0.012)		
2012–2015	0.425***	-0.047***	-0.096***	-0.144***	-0.185***	0.08	77,724
	(0.006)	(0.004)	(0.005)	(0.005)	(0.026)		
**Panel C:** Five-Year Sub-Samples							
2002–2007	0.202***	-0.102***	-0.174***	-0.235***	-0.345***	0.35	129,540
	(0.007)	(0.006)	(0.006)	(0.007)	(0.008)		
2004–2009	0.374***	-0.122***	-0.190***	-0.216***	-0.299***	0.55	129,540
	(0.006)	(0.006)	(0.006)	(0.006)	(0.008)		
2006–2011	0.383***	-0.097***	-0.172***	-0.211***	-0.264***	0.46	129,540
	(0.006)	(0.005)	(0.005)	(0.006)	(0.007)		
2008–2013	0.093***	-0.067***	-0.125***	-0.171***	-0.178***	0.60	129,540
	(0.006)	(0.006)	(0.006)	(0.007)	(0.011)		
2010–2015	0.213***	-0.056***	-0.113***	-0.165***	-0.181***	0.22	129,540
	(0.005)	(0.0045	(0.005)	(0.006)	(0.019)		

*N*ote: Clustered standard-errors are reported in parentheses. Year fixed-effects are included. ** and *** indicate statistical significance at the 5% and 1% levels, respectively.

There are two sub-samples the coefficients $\delta_{4}$ to $\delta_{\text{1}}$ are not increasingly negative: the 2012–2013 and the 2010–2013 sub-samples. The inconsistency lies within $\delta_{2}$ and $\delta_{\text{1}}$ whereby 2-star funds underperform the 1-star funds. The coefficient estimates of the 2- and 1-star funds are -0.123 and -0.100, respectively, for the 2012–2013 sub-sample and -0.172 and -0.152, respectively, for the 2010–2013 sub-sample. Overall, this represents an underperformance of the 2-star funds relative to the 1-star funds of approximately 2%. This may be attributed to market volatility occurring during these sub-sample periods as a result of the EDC. It should be noted that the risk calculations of ratings based on EU-E (*λ* = 0.25) can be attributed a higher weighting towards expected utility relative to entropy [31]. Therefore, the foregoing of a higher weighting towards entropy during a volatile market climate could be a plausible explanation for this inconsistency. We further explore this possibility in the following sections.

Interestingly, the abovementioned inconsistency cannot be detected when using the other three out-of-sample performance measures. Rather, 2-star funds for the 2014–2015 and 2012–2015 sub-samples contradict patterns of an increasingly negative relationship among the star rating groups. More specifically, for the 2014–2015 sub-sample, 2-star funds significantly outperform 5-star funds by 4.5% when using Jensen’s alpha as an out-of-sample performance measure. In the same sub-sample, we find no significant difference in the performance of the 2- and 5-star funds when using the alpha from the Fama-French 3-factor model as a performance measure, and 2-star funds outperform 3-star funds when using the alpha from the Carhart 4-factor model to measure fund performance. Furthermore, for the 2012–2015 sub-sample, 3-star funds outperform 4-star funds, and 2-star funds outperform both 3- and 4-star funds when using alphas from the single-, three-, and four-factor models to measure out-of-sample performance.

#### 4.1.3 Predictive ability of ratings based on EU-E (λ = 0.75)

Similar to the fund rating using EU-E (*λ* = 0.25) approach, we perform the fund ratings based on EU-E model when *λ* = 0.75 on the all 2159 funds at each month during different sample periods. The reason to chose *λ* = 0.75 is that it is the third quartile of the range of *λ* which is the proper trade-off between the expected utility and entropy indicating a higher weight of uncertainty of the return of the fund for an investor.

We present results derived from the panel regressions where independent variables are constructed using the ratings based on EU-E (*λ* = 0.75) and where the dependent variable is the Sharpe ratio. The Sharpe ratio is calculated one month in advance of rating using returns for each fund 3 years prior to the month evaluated. Each sub-sample period begins in August and ends in July of the denoted year. Clustered standard-errors are reported in parentheses. Year fixed-effects are included. Panels A, B, and C illustrate the one-, three-, and five-year sub-samples, respectively.

In contrast to that of ratings based on the EU-E model where *λ* is 0.25, the predictive ability of ratings based on EU-E (*λ* = 0.75) is largely inconsistent with our explanations and diminishes across the median and lower rating categories. We find that ratings based on EU-E (*λ* = 0.75) can predict top-rated funds, earning positive and significant risk-adjusted returns across all sub-samples. This is observed across all samples, though not for the 2002–2003 and 2008–2013 samples where $\delta_{5}$ is 0.003 and insignificant and where -0.100 and significant, respectively. We also observe negative and significant coefficients for $\delta_{4}$ across all sub-samples, showing that 4-star funds perform worse than the 5-star funds.

However, when assessing the predictive ability of the median and lower rated groups, no consistent pattern clearly shows that one rating group performs better or worse than another. As is shown in Panels A, B, and C of Table 6, no improvement is observed in the predictive ability of the ratings based on EU-E (*λ* = 0.75) over longer time horizons. The inability for these ratings to consistently predict future fund performance is robust to the use of the other three out-of-sample performance measures.

**Table 6:** Panel regression results derived from ratings based on EU-E (*λ* = 0.75) where the Sharpe ratio measures out-of-sample performance.

	$\delta_{5}$	$\delta_{4}$	$\delta_{3}$	$\delta_{2}$	$\delta_{1}$	$\mathrm{Adj}.R^{2}$	Obs.
**Panel A:** One-Year Sub-Samples							
2002–2003	0.003	-0.045***	0.027**	0.058***	0.101***	0.05	25,908
	(0.007)	(0.011)	(0.011)	(0.012)	(0.019)		
2004–2005	0.238***	-0.053***	-0.055***	-0.035***	-0.028***	0.07	25,908
	(0.006)	(0.007)	(0.008)	(0.007)	(0.010)		
2006–2007	0.454***	-0.085***	-0.198***	-0.347***	-0.503***	0.60	25,908
	(0.009)	(0.009)	(0.010)	(0.010)	(0.013)		
2012–2013	0.288***	-0.025***	0.007	0.089***	0.152***	0.13	25,908
	(0.005)	(0.005)	(0.007)	(0.007)	(0.016)		
2014–2015	0.501***	-0.031***	-0.115***	-0.226***	-0.280***	0.12	25,908
	(0.006)	(0.006)	(0.007)	(0.008)	(0.038)		
**Panel B:** Three-Year Sub-Samples							
2002–2005	0.035***	-0.056**	-0.019**	0.017**	0.058***	0.15	77,724
	(0.006)	(0.007)	(0.008)	(0.008)	(0.011)		
2004–2007	0.356***	-0.069***	-0.147***	-0.238***	-0.324***	0.35	77,724
	(0.007)	(0.008)	(0.008)	(0.008)	(0.011)		
2006–2009	0.398***	-0.083***	-0.160***	-0.265***	-0.315***	0.57	77,724
	(0.006)	(0.006)	(0.006)	(0.006)	(0.008)		
2010–2013	0.069***	-0.024***	-0.010*	0.082***	0.155***	0.37	77,724
	(0.005)	(0.004)	(0.006)	(0.006)	(0.012)		
2012–2015	0.367***	-0.026***	-0.045***	-0.054***	-0.051**	0.03	77,724
	(0.006)	(0.005)	(0.006)	(0.007)	(0.023)		
**Panel C:** Five-Year Sub-Samples							
2002–2007	0.118***	-0.064***	-0.088***	-0.125***	-0.158***	0.19	129,540
	(0.007)	(0.007)	(0.007)	(0.007)	(0.009)		
2004–2009	0.350***	-0.074***	-0.145***	-0.232***	-0.283***	0.57	129,540
	(0.006)	(0.005)	(0.006)	(0.006)	(0.008)		
2006–2011	0.328***	-0.062***	-0.110***	-0.153***	-0.140***	0.36	129,540
	(0.006)	(0.005)	(0.005)	(0.005)	(0.008)		
2008–2013	-0.100***	-0.033***	-0.026***	-0.034***	-0.105***	0.59	129,540
	(0.005)	(0.004)	(0.005)	(0.005)	(0.010)		
2010–2015	0.114***	-0.025***	-0.035***	-0.002	0.032*	0.17	129,540
	(0.005)	(0.004)	(0.005)	(0.006)	(0.017)		

*N*ote: Clustered standard-errors are reported in parentheses. Year fixed-effects are included. *, **, and *** indicate statistical significance at the 10%, 5% and 1% levels, respectively.

### 4.2 Predictive ability of fund ratings approaches during crises and recession periods

This section analyses the predictive abilities of the ratings based on Morningstar and the EU-E model during financial crises and economic recessions. Tables 7 and 8 report the results of the panel regressions where the independent variables are formed by using the ratings based on Morningstar and the EU-E model for crisis vs. non-crisis and recession vs. non-recession sub-samples, respectively.

**Table 7:** Panel regression results across crises sub-samples where the Sharpe ratio measures out-of-sample performance.

	$\delta_{5}$	$\delta_{4}$	$\delta_{3}$	$\delta_{2}$	$\delta_{1}$	$\mathrm{Adj}.R^{2}$	Obs.
**Panel A:** Global Financial Crisis							
EU-E	0.318***	-0.146***	-0.194***	-0.191***	-0.245***	0.42	41,021
(λ = 0)	(0.007)	(0.007)	(0.007)	(0.007)	(0.009)		
EU-E	0.332***	-0.128***	-0.210***	-0.229***	-0.288***	0.47	41,021
(λ = 0.25)	(0.006)	(0.006)	(0.006)	(0.006)	(0.009)		
EU-E	0.342***	-0.107***	-0.208***	-0.275***	-0.323***	0.56	41,021
(λ = 0.50)	(0.006)	(0.005)	(0.005)	(0.006)	(0.008)		
EU-E	0.322***	-0.096***	-0.170***	-0.269***	-0.280***	0.53	41,021
(λ = 0.75)	(0.006)	(0.006)	(0.006)	(0.006)	(0.009)		
EU-E	0.331***	-0.098***	-0.170***	-0.277***	-0.308***	0.56	41,021
(λ = 1)	(0.006)	(0.006)	(0.005)	(0.006)	(0.008)		
Morningstar Ratings	0.246***	-0.047***	-0.101***	-0.152***	-0.223***	0.39	41,021
	(0.009)	(0.009)	(0.009)	(0.009)	(0.011)		
**Panel B:** European Debt Crisis							
EU-E	0.222***	-0.068***	-0.148***	-0.227***	-0.257***	0.41	41,021
(λ = 0)	(0.007)	(0.008)	(0.008)	(0.008)	(0.009)		
EU-E	0.180***	-0.048***	-0.112***	-0.172***	-0.167***	0.29	41,021
(λ = 0.25)	(0.007)	(0.008)	(0.008)	(0.009)	(0.012)		
EU-E	0.121***	-0.031***	-0.059***	-0.092***	-0.124*	0.20	41,021
(λ = 0.50)	(0.006)	(0.006)	(0.007)	(0.007)	(0.013)		
EU-E	0.059***	-0.030***	-0.033***	0.045***	0.134***	0.30	41,021
(λ = 0.75)	(0.004)	(0.005)	(0.005)	(0.007)	(0.011)		
EU-E	0.057***	0.001	0.012*	0.011	0.052***	0.18	41,021
(λ = 1)	(0.004)	(0.006)	(0.006)	(0.007)	(0.010)		
Morningstar Ratings	0.149***	-0.056***	-0.083***	-0.010***	-0.145***	0.20	41,021
	(0.013)	(0.013)	(0.013)	(0.014)	(0.016)		
**Panel C:** Non-Crisis							
EU-E	0.184***	-0.090***	-0.153***	-0.214***	-0.289***	0.40	254,762
(λ = 0)	(0.006)	(0.004)	(0.004)	(0.005)	(0.010)		
EU-E	0.171***	-0.081***	-0.145***	-0.200***	-0.269***	0.38	254,762
(λ = 0.25)	(0.005)	(0.004)	(0.004)	(0.004)	(0.011)		
EU-E	0.127***	-0.050***	-0.103***	-0.143***	-0.189***	0.33	254,762
(λ = 0.50)	(0.005)	(0.004)	(0.004)	(0.004)	(0.012)		
EU-E	0.083***	-0.045***	-0.061***	-0.073***	-0.072***	0.28	254,762
(λ = 0.75)	(0.005)	(0.004)	(0.004)	(0.004)	(0.011)		
EU-E	0.085***	-0.031***	-0.050***	-0.096***	-0.112***	0.30	254,762
(λ = 1)	(0.005)	(0.004)	(0.005)	(0.005)	(0.011)		
Morningstar Ratings	0.110***	-0.058***	-0.085***	-0.119***	-0.185***	0.30	254,762
	(0.007)	(0.006)	(0.006)	(0.006)	(0.016)		

*Note*: Clustered standard-errors are reported in parentheses. Year fixed-effects are included. * and *** indicate statistical significance at the 10% and 1% levels, respectively.

**Table 8:** Panel regression results across recession sub-samples where the Sharpe ratio measures out-of-sample performance.

	$\delta_{5}$	$\delta_{4}$	$\delta_{3}$	$\delta_{2}$	$\delta_{1}$	$\mathrm{Adj}.R^{2}$	Obs.
**Panel A:** Recession							
EU-E	0.192***	-0.121***	-0.162***	-0.180***	-0.221***	0.39	41,021
(λ = 0)	(0.006)	(0.006)	(0.007)	(0.007)	(0.007)		
EU-E	0.199***	-0.111***	-0.174***	-0.197***	-0.239***	0.42	41,021
(λ = 0.25)	(0.005)	(0.005)	(0.005)	(0.006)	(0.007)		
EU-E	0.197***	-0.091***	-0.169***	-0.212***	-0.236***	0.46	41,021
(λ = 0.50)	(0.005)	(0.005)	(0.005)	(0.006)	(0.008)		
EU-E	0.170***	-0.085***	-0.130***	-0.171***	-0.181***	0.38	41,021
(λ = 0.75)	(0.005)	(0.005)	(0.005)	(0.006)	(0.009)		
EU-E	0.174***	-0.083***	-0.127***	-0.193***	-0.200***	0.40	41,021
(λ = 1)	(0.005)	(0.005)	(0.005)	(0.006)	(0.008)		
Morningstar Ratings	0.140***	-0.054***	-0.101***	-0.142***	-0.205***	0.35	41,021
	(0.008)	(0.008)	(0.008)	(0.008)	(0.010)		
**Panel B:** Non-Recession							
EU-E	0.186***	-0.090***	-0.157***	-0.218***	-0.288***	0.38	295,783
(λ = 0)	(0.006)	(0.004)	(0.004)	(0.005)	(0.009)		
EU-E	0.170***	-0.079***	-0.145***	-0.201***	-0.261***	0.36	295,783
(λ = 0.25)	(0.005)	(0.004)	(0.004)	(0.004)	(0.010)		
EU-E	0.126***	-0.049***	-0.102***	-0.145***	-0.176***	0.30	295,783
(λ = 0.50)	(0.005)	(0.004)	(0.004)	(0.004)	(0.011)		
EU-E	0.081***	-0.045***	-0.063***	-0.068***	-0.057***	0.26	295,783
(λ = 0.75)	(0.005)	(0.004)	(0.004)	(0.004)	(0.010)		
EU-E	0.083***	-0.029***	-0.047***	-0.104***	-0.098***	0.27	295,783
(λ = 1)	(0.005)	(0.004)	(0.004)	(0.005)	(0.010)		
Morningstar Ratings	0.109***	-0.057***	-0.085***	-0.118***	-0.182***	0.17	295,783
	(0.007)	(0.006)	(0.006)	(0.006)	(0.015)		

*Note*: Clustered standard-errors are reported in parentheses. *** indicates statistical significance at the 1% level.

Panel A of Table 7 presents the results during the GFC period where the Sharpe ratio measures out-of-sample performance. We find a significant and increasingly negative relationship across coefficients $\delta_{\text{4}}$ to $\delta_{\text{1}}$ for all funds ranked by Morningstar and all EU-E models. Consistent with our expectations, there is an improvement in the predictive ability of the ratings based on the EU-E model as λ approaches 1, demonstrated by the increasingly negative relationship from $\delta_{\text{4}}$ to $\delta_{\text{1}}$. This finding is consistent with prior literature that entropy is able to successfully capture the effects of market volatility [44].

The performance of the ratings calculated using EU-E (λ = 0.75) and EU-E (λ = 1) diminishes during the EDC. As reported in Panel B of Table 7, the coefficients on $\delta_{\text{2}}$ and $\delta_{\text{1}}$ for the ratings based on EU-E (λ = 0.75) are 0.045 and 0.134, respectively during the EDC, indicating that the 2- and 1-star funds outperform the 5-star funds over this period. As λ increases to 1, the ratings based on the EU-E model become less efficient in their predictive ability as the coefficients on $\delta_{\text{4}}$, -0.001, and $\delta_{\text{2}}$, 0.011, are positive and insignificant. Additionally, the coefficients on $\delta_{\text{3}}$, 0.012, and $\delta_{\text{1}}$, 0.052, are positive and significant. During the non-crisis sub-samples, a decline in performance of the ratings based on EU-E (λ = 0.75) and EU-E (λ = 1) is evidenced by a fall in the average differences between star rating groups throughout the non-crisis period. A fall in the average differences between star rating groups suggests a decline in predictive ability as this demonstrates that the model is less able to accurately rank funds into appropriate star categories. For instance, as λ moves from 0 to 1, the coefficient estimates for $\delta_{\text{5}}$ through to $\delta_{\text{1}}$ using the ratings calculated by EU-E (λ = 0) are 0.184, -0.090, -0.153, -0.214, and -0.289, respectively, and the ratings based on EU-E (λ = 0.25) are 0.171, -0.081, -0.145, -0.200 and -0.269, respectively. This pattern of declining magnitudes on each coefficient continues through the ratings based on the EU-E model as λ approaches 1. Similar results to the GFC and non-crisis periods are shown in the recession and non-recession subsamples, respectively, in Table 8.

The performance of Morningstar ratings over crisis vs. non-crisis and recession vs. non-recession periods is consistent with the results reported in Table 4. To differentiate between the performance of Morningstar ratings and the ratings based on the EU-E model, we examine the relative magnitudes on the coefficients of each rating group. Two main findings are observed. Firstly, during periods of strong market volatility (i.e. the GFC and recession sub-samples), the ratings based on Morningstar underperforms each of the EU-E model based ratings. For example, the average risk-adjusted returns earned by the 5-star funds based on EU-E (λ = 0.25) during the GFC sub-sample is 0.332 whereas Morningstar’s 5-star funds over the same period is only 0.246. This result is consistent across all λ values (i.e. where λ takes a value of 0, 0.25, 0.50, 0.75, and 1) and star categories during the GFC and recession sub-samples. Secondly, during less volatile market conditions, funds ranked by Morningstar ratings underperform those ranked by the EU-E model with a lower λ value (i.e. 0.25 and 0), but outperform those with a higher λ value (i.e. 0.75 and 1).

Overall, these results support that the EU-E decision model provides a greater level of predictive ability than Morningstar ratings during crisis and non-crisis periods, however this result is confined to λ values less than 0.50.

### 4.3 Comparison of predictive abilities across rating models

We present a summary of the predictive ability of the ratings derived from Morningstar and the EU-E model where *λ* = 0, 0.25, 0.50, 0.75, and 1, across each performance metric (i.e., the Sharpe Ratio, Jensen’s Alpha, the alpha from the Fama-French 3-Factor Model, and the Carhart 4-Factor Model). The total number of regressions performed for each performance metric is 20, which accounts for each sub-sample considered in the overall sample. A significant negative relationship is defined as an increasingly negative relationship among the coefficients from $\delta_{4}$ to $\delta_{\text{1}}$ such that $0>\delta_{\text{4}}>\delta_{\text{3}}>\delta_{\text{2}}>\delta_{\text{1}}$.

Overall, we find that Morningstar ratings offer superior predictive ability as defined by an increasingly negative relationship of the coefficients $\delta_{4}$ to $\delta_{\text{1}}$ across all regressions. The predictive ability of the ratings based on the EU-E model is strongest when *λ* is 0 and is weakest when *λ* is 0.75. In general, the predictive ability of ratings based on the EU-E model declines as *λ* approaches 1. This further shows that a lower trade-off coefficient (*λ*) is more stable and efficient than a larger value. As is shown in Panel B of Table 9, the ratings based on Morningstar and the EU-E model perform well when applied during crisis and recession periods with the exception of the EU-E (*λ* = 0.75) model. A possible explanation for this result may be the characteristics of the sample considered in this study. As the S&P 500 during the overall sample undergoes steady market appreciation except for the GFC, returns of the funds should be fairly stable across time. Therefore, as entropy in the EU-E model considers the risk of volatility in a fund’s returns [35], greater consideration for entropy during stable market conditions would reduce the efficiency of the risk measure used in the EU-E model.

**Table 9:** Summary of the predictive ability of rating measures observed across performance metrics.

Number of Times Predictor Produces a Significant Negative Relationship Using the Performance Metric				
Predictor	Sharpe Ratio	Jensen’s Alpha	Fama-French 3-Factor Model	Carhart 4-Factor Model
**Panel A:** Overall Sample				
Morningstar Ratings	20	20	20	20
EU-E (*λ* = 0)	18	19	19	19
EU-E (*λ* = 0.25)	17	18	18	18
EU-E (*λ* = 0.50)	15	13	13	13
EU-E (*λ* = 0.75)	7	3	3	5
EU-E (*λ* = 1)	11	12	10	11
**Panel B:** Crisis and Recession Periods				
Morningstar Ratings	3	3	3	3
EU-E (*λ* = 0)	2	3	3	3
EU-E (*λ* = 0.25)	2	3	3	3
EU-E (*λ* = 0.50)	2	3	3	3
EU-E (*λ* = 0.75)	1	1	1	2
EU-E (*λ* = 1)	2	3	2	3

To examine the comparative performance of the 5-star category, we assess the number of times the 5-star rating category based on the EU-E models outperforms the 5-star rating categories developed by Morningstar such that $\delta_{5,EU-E}>\delta_{5,MORNINGSTAR}$. As investors seek to invest in funds providing the highest risk-adjusted returns, a higher coefficient on $\delta_{5}$ is preferred. To assess the comparative performance of rating categories 4 to 1, we assess the number of times the EU-E model-based rating category outperforms relative to the Morningstar rating category. This approach is used because coefficients on the dummy variables represent the underperformance of the star category relative to the 5-star category. Thus, the presence of a greater difference between each star category indicates a greater level of distinction in the performance of each star category. As such, a greater level of outperformance of the star categories 4 to 1 relative to the preceding star category is preferred. The comparative outperformance of an EU-E model rated 3-, 2-, or 1-star category relative to that of Morningstar ratings is defined as the existence of positive difference in the star category relative to the preceding star category, compared to that of Morningstar ratings. For example, the outperformance of a 3-star group based on EU-E model relative to a Morningstar 3-star group is defined as the existence of the inequality holding such that: $\left(\delta_{4,EU-E}-\delta_{3,EU-E})>(\delta_{4,MORNINGSTAR}-\delta_{3,MORNINGSTAR}\right)$.

We present a comparative summary of the predictive ability of the ratings based on EU-E (*λ* = 0.25) and EU-E (*λ* = 0.75) with Morningstar ratings reported across each of the out-of-sample performance measures in Table 10. The total number of regressions performed accounting for each sub-sample, and each performance metric is 80 in Panel A and 12 in Panel B.

**Table 10:** Comparison of the predictive ability of ratings based on the EU-E model with Morningstar ratings measured across performance metrics.

Star Rating Category	Number of Times EU-E 5-star rating category outperforms Morningstar 5-star rating category	Number of Times EU-E rating category outperforms Morningstar rating category			
		4-Star	3-Star	2-Star	1-Star
**Panel A:** Overall Sample					
EU-E (*λ* = 0.25)	69	58	50	41	47
EU-E (*λ* = 0.75)	48	63	35	10	8
**Panel B:** Crisis and Recession Periods					
EU-E (*λ* = 0.25)	12	11	7	4	1
EU-E (*λ* = 0.75)	12	11	10	2	0

Panel A of Table 10 shows that both ratings based on the EU-E model are superior to the Morningstar ratings in forecasting the top performing funds. In particular, ratings based on EU-E (*λ* = 0.25) predict the top performing funds better than Morningstar ratings on 69 of the 80 occasions. The 4-star category of ratings calculated using EU-E (*λ* = 0.25) shows a high levels of outperformance relative to its 5-star group in comparison to Morningstar ratings, outperforming relative to Morningstar in 58 of the 80 regressions. However, the outperformance of each star category based on EU-E (*λ* = 0.25) relative to Morningstar ratings declines steadily to the point of being roughly on par with Morningstar ratings when examining the median and lower rated categories. The improved performance of Morningstar ratings observed when examining lower rated funds is consistent with the findings of prior studies [8] [13–15]. This result may be attributed to the concavity of the utility curve used by Morningstar, which is derived from the EUT. The concave utility curve places more emphasis on downward variations of returns within a particular fund category, thus providing Morningstar with greater differential ability of the lower rated funds relative to the higher rated funds [37]. In other words, funds with a lower expected utility (i.e., lower rated funds) face a stricter penalty than funds with a higher expected utility (i.e., higher rated funds), and thus the EUT naturally provides Morningstar ratings with greater differential ability of the lower rated funds than higher rated funds. This is consistent with Lisi and Caporin [17], who show that Morningstar’s constant relative risk aversion coefficient (*γ*) of 2 fails to capture the preferences of all investors.

A similar pattern is observed when examining the performance of ratings based on EU-E (*λ* = 0.75) relative to those of Morningstar. Ratings calculated using EU-E (*λ* = 0.75) outperform Morningstar ratings in predicting the best performing funds in 48 of the 80 occasions. This improves to 63 of all 80 occasions when examining the outperformance of 4-star ratings relative to 5-star ratings based on EU-E (*λ* = 0.75) relative to those of Morningstar. However, the performance of the EU-E (*λ* = 0.75) based ratings strongly deteriorates when examining the median and lower rated categories.

Panel B of Table 10 reports similar relationships between ratings based on the EU-E (*λ* = 0.25) and EU-E (*λ* = 0.75) models relative to those of Morningstar during crisis and recession periods. Both EU-E models outperform Morningstar in differentiating the 5- and 4-star categories 12 and 11 times out of the 12 occasions, respectively. Consistent with Panel A of Table 10, Morningstar ratings perform better for the lower rated categories, outperforming the 1-star funds of both EU-E models in almost all regressions.

Overall, both ratings based on the EU-E model where *λ* takes values of 0.25 and 0.75 tend to predict the top performing funds better than Morningstar ratings. However, the performance of the EU-E model-based ratings declines when assessing the outperformance of the lower rating categories relative to the corresponding rating category used in Morningstar ratings. Ratings based on EU-E (*λ* = 0.25) is a superior measure than that of Morningstar. Complementary to the results shown in Table 6, where we find that the EU-E (*λ* = 0.25)-based ratings offer superior predictive ability among rating groups, Table 10 shows that ratings based on EU-E (*λ* = 0.25) demonstrate a higher level of predictive ability in forecasting the top performing funds and can better differentiate the performance between star categories. Ratings based on EU-E (*λ* = 0.75) does not offer superior predictive ability when applied to each star category, while it is better at selecting the top performing funds than Morningstar. Thus, if only the highest rating categories are indeed relevant to an investor, ratings based on the EU-E model where *λ* takes values of 0.25 and 0.75 will be preferred to Morningstar ratings. On the contrary, the EU-E model (λ = 0.75) underperforms Morningstar in predicting the worst performing funds. This could be explained by risk measure introduced in the EU-E model. Fund ratings may be affected if the value of λ is assigned improperly. For example, when returns are relatively small, which is the case of the lower rated funds, the expected utility of the fund will have less weight (1- λ) if a higher λ is assigned. Hence, the measure of the uncertainty of returns will have a greater influence on lower rated funds relative to those with a higher rating. As the result, the predictive ability of EU-E model compared to Morningstar ratings performs worse for the lower rated funds. This result can be further explained by Morningstar’s reliance on the EUT. The utility curve with respect to the EUT is defined by γ, which Morningstar assigns a value of 2 corresponding to a risk averse investor. This results in a concave utility curve which places greater emphasis on downward variations in returns which allows Morningstar to more effectively differentiate the worst performing, as opposed to the best performing funds.

## 5 Conclusions

Mutual funds have become an increasingly dominant industry and asset class within financial markets in recent times. This paper examines one of the most widely renowned fund rating tools used in the industry, Morningstar ratings, with respect to a newly developed decision tool, the EU-E decision model, which is also used to rank financial assets. We explore the EU-E decision model as a possible alternative given its ability to potentially mitigate drawbacks of the risk measure used in Morningstar ratings.

Prior literature shows that Morningstar ratings lack predictive ability when examining the future performance of higher and median rated funds and that this may be due to the absence of a suitable risk measure inherent to the model [8], [12–15], [17]. Thus, we propose the EU-E decision model as a suitable alternative given its proven ability to solve simple decision problems found to contradict the EUT and also for its inherent consideration for investor behaviour [31].

Overall, we find that ratings based on the EU-E (*λ* = 0.25) and EU-E (*λ* = 0.75) models outperform the Morningstar ratings in predicting the best performing funds. However, this predictive ability declines across rating groups. This finding is consistent with prior literature showing that Morningstar ratings is able to predict the worst rather than the best performing funds [8], [12–15]. With respect to the predictive ability of ratings based on the EU-E model, we show that a lower trade-off coefficient (*λ*) generates more efficient results, and this is consistent with the EU-E model’s ability to effectively consider investor preferences under risk [31].

We checked the sensitivity of our results across different holding periods for out-of-sample performance, different rolling windows and methods from the prior literature by conducting cross sectional dummy variable regressions and these findings are robust to these robustness checks. Given our findings, we conclude that ratings based on the EU-E (*λ* = 0.25) model is the best performing measure as proven by its superior near- to long-term predictive ability, which holds across volatile and stable markets.

Importantly, we find that the measure of risk used in the calculation of ratings plays an integral role in the performance of the rating models. We find evidence that the EU-E model is able to effectively consider an investor’s decisions under risk. However, this result only holds when applying a lower *λ* value.

In summary, we conclude that the fund ratings based on the EU-E model (*λ* = 0.25, 0.75) outperform (underperform) Morningstar ratings in predicting higher (lower) rated funds. However, this predictive ability declines across rating groups. From an economic point of view, the results suggest that the fund ratings approach based on the EU-E model has the potential to assist investors in selecting the best performing funds, however, it may not be the most efficient approach in identifying the worst performing funds. This may affect the efficient allocation of capital by investors relying on the EU-E model as it has the potential to correctly direct fund flow for higher rated funds, but not for lower rated funds as there is an evidence of inflows to funds gaining stars and outflows to funds losing stars [9]. Usually, a rational investor, who seeks to generate positive returns, would invest in higher rather than lower rated funds given that the short positions on mutual funds are generally not possible. Thus, it could have almost no significant effect to the investment decision for a rational investor even though fund ratings based on EU-E models underperforms Morningstar ratings in predicting the worst performing funds.

## Supporting information

S1 Table Monthly returns for all U.S. mutual funds. (XLSX)

S2 Table Morningstar overall rating for all U.S. mutual funds. (XLSX)

S3 Table Factors used to calculate mutual funds’ performance. (XLSX)

## References

[1] Khorana A, Servaes H (2012), What drives market share in the mutual fund industry? Rev Financ 16: 81–113.

[2] ICI (2016), Investment Company Fact Book. Investment Company Institute. 3 Jun 2016. Available from: https://www.ici.org/pdf/2016_factbook.pdf

[3] Wilcox R (2003), Bargain hunting or star gazing? Investors’ preferences for stock mutual funds. J Bus 76: 645–663.

[4] French K (2008), Presidential address: The cost of active investing. J Finan 63: 1537–1573.

[5] Capon N, Fitzsimons G, Prince R (1996), An individual level analysis of the mutual fund investment decision. J Financ Serv Res 10: 59–82.

[6] Adkisson J, Fraser D (2003), Reading the stars: age bias in Morningstar ratings. Financ Anal J 59: 24–27.

[7] Graham E, Lassala C, Ribeiro-Navarrete B (2018), A fuzzy-set analysis of conditions influencing mutual fund performance. Int Rev Econ Financ. 10.1016/j.iref.2018.01.017

[8] Blake C, Morey M (2000), Morningstar ratings and mutual fund performance. J Finan Quant Anal 35: 451–483.

[9] Del Guercio D, Tkac P (2008), Star power: The effect of Morningstar ratings on mutual fund flow. J Finan Quant Anal 43: 907–936.

[10] Phillips B, Pukthuanthong K, Rau R (2016), Past performance may be an illusion: performance flows and fees in mutual funds. Crit Finan Rev 5: 351–398.

[11] Kaniel R, Parham R (2017), WSJ category kings–The impact of media attention on consumer and mutual fund investment decisions. J Finan Econ 123: 337–356.

[12] Sharpe W (1998), Morningstar’s risk-adjusted ratings. Financ Anal J 54: 21–33.

[13] Gerrans P (2006), Morningstar ratings and future performance. Account Financ 46: 605–628.

[14] Füss R, Hille J, Rindler P, Schmidt J, Schmidt M (2010), From rising stars and falling angels: On the relationship between the performance and ratings of German mutual funds. J Wealth Manag 13: 75–90.

[15] Sah V, Tidwell O, Ziobrowski A (2011), The predictive abilities and persistence of Morningstar ratings: An examination of real estate mutual funds. J Prop Res 28: 249–267.

[16] Watson J, Delaney J, Dempsey M, Wickramanayake J (2016), Australian superannuation Pension fund product ratings and performance: A guide for fund managers. Aust J Manag 41: 189–211.

[17] Lisi F, Caporin M (2012), On the role of risk in the Morningstar rating for mutual funds. Quant Financ 12: 1477–1486.

[18] Allais M (1953), Le comportement de l’Homme rationnel devant le risque: Critique des postulats et axiomes de l’ecole Americaine. Econometrica 21: 503–546.

[19] Kahneman D, Tversky A (1979), Prospect theory: An analysis of decision under risk. Econometrica 47: 263–291.

[20] Rabin M (2000), Risk aversion and expected-utility theory: A calibration theorem. Econometrica 68: 1281–1292.

[21] Niendorf B, Ottaway T (2002), Wealth effects of time variation in investor risk preferences. J Econ Financ 26: 77–87.

[22] Shannon C (1948), A mathematical theory of communication. Bell Syst Tech J 27: 379–423+623–656.

[23] Buchen P, Kelly M (1996), The maximum entropy distribution of an asset inferred from option prices. J Finan Quant Anal 31: 143–159.

[24] Maasoumi E, Racine J (2002), Entropy and predictability of stock market returns. J. Econometrics 107: 291–312.

[25] Caraiani P (2014), The predictive power of singular value decomposition entropy for stock market dynamics. Phys A 393: 571–578.

[26] Philippatos G, Wilson C (1972), Entropy, market risk, and the selection of efficient portfolios. Appl Econ 4: 209–220.

[27] Philippatos G, Gressis N (1975), Conditions of equivalence among E-V, SSD, and E-H portfolio selection criteria: The case for uniform, normal and lognormal distributions. Manage Sci 21: 617–625.

[28] Dionisio A, Menezes R, Mendes D (2006), An econophysics approach to analyse uncertainty in finance markets: an application to the Portuguese stock market. Eur Phys J B 50: 161–164.

[29] Bentes S, Menezes R (2012), Entropy: A new measure of stock market volatility? J Phys Conf Ser 394: 1–6(012033).

[30] Ormos M, Zibriczky D (2014), Entropy-based financial asset pricing. PLoS ONE 9: e115742 10.1371/journal.pone.0115742 25545668 PMC4278763

[31] Yang J, Qiu W (2005), A measure of risk and a decision-making model based on expected utility and entropy. Eur J Oper Res 164: 792–799.

[32] Luce RD, Ng CT, Marley A, Aczél J (2008a), Utility of gambling I: entropy modified linear weighted utility. Econ Theory 36: 1–33.

[33] Luce RD, Ng CT, Marley A, Aczél J (2008b), Utility of gambling II: risk, paradoxes, and data. Econ Theory 36: 165–187.

[34] Yang J, Qiu W (2014), Normalized expected utility-entropy measure of risk. Entropy 16: 3590–3604.

[35] Yang J, Feng Y, Qiu W (2017), Stock selection for portfolios using expected utility-entropy decision model. Entropy 19: 508.

[36] Bechmann K, Rangvid J (2007), Rating mutual funds: Construction and information content of an investor-cost based rating on Danish mutual funds. J Empir Financ 14: 662–693.

[37] Morningstar (2009), The Morningstar rating methodology. Morningstar. 28 March 2016. Available from: http://corporate.morningstar.com/US/documents/MethodologyDocuments/FactSheets/MorningstarRatingForFunds_FactSheet.pdf

[38] Sharpe W (1966), Mutual fund performance. J Bus 39: 119–138.

[39] Fama E, French K (1993), Common risk factors in the returns on stocks and bonds. J Finan Econ 33: 3–56.

[40] Carhart M (1997), On persistence in mutual fund performance. J Finan 52: 57–82.

[41] Morey M, Gottesman A (2006), Morningstar mutual fund ratings redux. J Invest Consult 8: 25–37.

[42] Morey M (2002), Mutual fund age and Morningstar ratings. Financ Anal J 58: 56–63.

[43] Eling M, Schuhmacher F (2007), Does the choice of performance measure influence the evaluation of hedge funds. J Bank Finan 31: 2632–2647.

[44] Huang J, Shang P, Zhao X (2012), Multifractal diffusion entropy analysis on stock volatility in financial markets. Phys A 391: 5739–5745.

